# Household food insecurity is associated with a higher burden of obesity and risk of dietary inadequacies among mothers in Beirut, Lebanon

**DOI:** 10.1186/s12889-017-4317-5

**Published:** 2017-06-12

**Authors:** Lamis Jomaa, Farah Naja, Ruba Cheaib, Nahla Hwalla

**Affiliations:** Department of Nutrition and Food Sciences, Faculty of Agricultural and Food Sciences, American University of Beirut, P.O. Box 11-0.236 , Riad El Solh, Beirut, 11072020 Lebanon

**Keywords:** Household food insecurity, Obesity, Dietary intake, Women, Urban, Lebanon

## Abstract

**Background:**

Mixed evidence exists with respect to the association between household food insecurity (HFIS) and obesity in low-to-middle income countries (LMICs), particularly among women. This study aimed to measure socioeconomic correlates of HFIS and explores its association with dietary intake and odds of obesity among mothers in Lebanon, a middle-income country undergoing nutrition transition.

**Methods:**

A cross-sectional study was conducted among a representative sample of households (*n* = 378) in Beirut, Lebanon. Surveys were completed with mothers of children <18 years. HFIS was measured using a locally-validated, Arabic-translated Household Food Insecurity Access Scale (HFIAS). Dietary intake was assessed using the multiple pass 24-h recall method. Associations between HFIS (food vs food insecure) and socio-demographic characteristics were reported using crude and adjusted odds ratios. The odds of consuming <2/3rd Dietary Reference Intakes (DRIs) for nutrients among mothers from food secure and food insecure households were explored. In addition, logistic regression analyses were conducted to explore the association of HFIS with obesity (BMI ≥ 30 kg/m2) and at-risk waist circumference (WC ≥ 80 cm) among mothers.

**Results:**

HFIS was found among 50% of study sample and was inversely associated with household income and mother’s educational level, even after adjusting for other socioeconomic variables (*p* < 0.01). Mothers in food insecure households reported consuming significantly less dairy products, fruits, and nuts yet more breads and sweets; and they had higher odds of consuming <2/3rd the DRI’s for key micronutrients (potassium, folate, and vitamin C) compared to secure ones. Adjusting for socioeconomic correlates, food insecure mothers had 1.73 odds of obesity (95% CI: 1.02–2.92) compared to food secure mothers.

**Conclusions:**

High HFIS prevalence was reported among urban Lebanese households. Mothers from food insecure households had a high risk of dietary inadequacy and obesity. Adequate evidence-based public health strategies are needed to reduce the vulnerability of mothers to food insecurity in LMIC settings and alleviate their risk of a high burden of nutrient insecurity and obesity.

## Background

Food and nutrition insecurity are serious public health challenges affecting millions of households globally [1]. According to the 2001 Food and Agriculture Organization (FAO) definition, food insecure households and individuals have limited availability, access, and utilization of appropriate, safe, and nutritious food to meet their dietary needs and food preferences for an active and healthy life [1]. Household food insecurity (HFIS) can adversely affect the nutritional status and health of at risk groups, particularly women of reproductive age. Recent studies highlight that mothers living in food insecure households are at higher risk of inadequate dietary intake and are likely to adopt risky coping mechanisms, including limiting food intake and compromising quality of diet, to ensure children are being well-fed [2–4]. Low food security not only reduces the quality and variety of mothers’ diets, but can also compromise their nutritional status leading to deficiencies in beneficial micronutrients [4, 5]. Additionally, food insecurity has been associated with poor pregnancy outcomes, including preterm births, low birth weight, and gestational diabetes [6]. Early prenatal and postnatal metabolic conditions can have long-lasting effects that predispose the developing fetus to increased body weight and related comorbidities during later stages of life [7].

Food insecurity is a global challenge, particularly in LMICs in the Middle East and North Africa (MENA) region that are reported to be among the most food insecure worldwide [8]. With the most recent *Arab Uprisings*, food insecurity has become more than ever a serious challenge that threatens the ability of affected nations to provide food as a basic human right for its populations. Lebanon, a middle-income country in the MENA region with a relatively fragile political, social, and economic system that increases its risk of food insecurity, has recently suffered the repercussions of these *Arab Spring* movements, including the Syrian war. The latter is one of the largest humanitarian crises in today’s world that have led to an unprecedented large influx of Syrian refugees through the borders of Lebanon further taxing the limited economic resources of the country and contributing to its political and social instability [8, 9]. With the escalating Syrian humanitarian crisis, international agencies operating in Lebanon have been mostly concerned with exploring the food security status of refugees [10]. However, the food security status of Lebanese households has not been adequately explored nor was the association between household food security and nutritional status of vulnerable groups, particularly mothers. To date, only two published studies in Lebanon, primarily validating the use of food security assessment tools showed that moderate to severe HFIS were as high as 34% and 42% among Lebanese households in the rural Bekaa region [11] and those in a semi-urban area in the South of Lebanon [12], respectively.

The use of experience-based food security scales have been shown to be valid in various LMICs worldwide to measure food insecurity at the individual and household-levels. These scales that collect the perceptions and experiences of a member of a household to different aspects of food insecurity have been found to be theory-based and were suggested to be necessary indicators for food security governance [13]. However, studies testing the use of such scales in exploring HFIS and its association with specific nutrition outcomes within LMICs, including those in the MENA region, remain insufficient.

In parallel to the heightened economic and social challenges threatening to compromise the food security status of Lebanese households, the country, as with other LMICs of the region, has witnessed a significant increase in obesity rates during the past decade coupled with existing micronutrient deficiencies among at-risk population groups, mainly children and women of reproductive age [14–18]. According to two nationally representative studies conducted in Lebanon, obesity prevalence among adult women increased by almost 50% (≥ 20 years) between 1997 and 2009 (19.3% to 28.8%, respectively) [15]. These rising obesity rates were partly explained by the early nutrition transition that has been witnessed among the Lebanese population with a higher consumption of low-nutrient, energy-dense foods and beverages coupled with increased sedentary behaviors.

Previous national studies that assessed obesity prevalence in Lebanon did not evaluate the potential associations between HFIS, nutrient deficiencies, and obesity. A growing body of evidence suggests that a positive association exists between HFIS and adult obesity, and these findings are consistently reported among women in high income countries [19]. However, this link has been less explored in LMICs undergoing nutrition transition with limited studies showing mixed results with respect to the association of HFIS and body weight among women and mothers [20–24]. In addition, fewer studies explored the association between HFIS and nutrient inadequacy among female caregivers within urban settings in LMICs [25, 26]. The association between body weight, food consumption behaviors, and food insecurity is complex. It may vary based on socioeconomic and environmental conditions, demographic characteristics, cultural dynamics, and various coping mechanisms that can be adopted to address food shortage; all of which are factors that warrant further exploration in LMICs to adopt much-needed strategies well-positioned to respond to the growing food security challenges.

In view of the limited scientific evidence exploring the association between food insecurity, dietary intake, and nutritional status of women from LMICs in the MENA region, this study aimed at (1) assessing the prevalence and socio-demographic correlates of food insecurity among a representative sample of households in an urban setting in Lebanon; (2) examining the effect of food insecurity on dietary intake among mothers in the study sample; and (3) exploring the association between food insecurity and the odds of obesity among mothers. Findings from this study would be of significance, particularly to similar LMIC settings, as they can assist in devising evidence-based policies and public health interventions that can tackle the double burden of food insecurity and over nutrition among high-risk population groups.

## Methods

### Study design and sampling methodology

This is a cross-sectional study that was conducted among a representative sample of Lebanese households from the Greater Beirut area in Lebanon between December 2014 and May 2015. Households, the primary sampling unit in the study, were drawn using stratified cluster sampling, whereby the strata were the 24 cadastral zones of Greater Beirut. Each cadastral zone was divided into clusters comprised of 100–150 households. Within the cluster, households were selected by systematic sampling, based on *probability proportional to size* technique using the Lebanese Central Administration of Statistics as a reference [27]. Sample size calculations showed that 368 subjects were needed to provide 95% confidence interval around a food security prevalence estimate of 42% with ±5% variation. The estimate of food security (42%) used in the sample size calculations was based on a previous investigation of food security in a semi-urban Lebanese setting [12]. For a household to be eligible, it had to include a mother of a child below 18 years of age, and the mother be present at the time of the interview. The decision to include mothers of children younger than 18 years of age was undertaken to explore the effect of HFIS on mothers, who are at the time of the study the caregivers of children in the household. Inclusion criteria for the mothers were: 1) Lebanese citizenship, 2) absence of chronic disease that may affect the dietary intake, body composition and/or the overall nutritional status of the mothers, and 3) not taking any medications that may interfere with the eating patterns or body weight. Mothers who were either pregnant or breastfeeding at the time of the interview were not included in the study given the physiologic, dietary, and lifestyle changes that occur during pregnancy and lactation and that may affect the dietary intake and nutritional status of women. Of those who accepted to participate in the study and met the eligibility criteria (*n* = 391), 388 mothers completed the interview.

### Data collection

Face-to-face interviews with study participants were conducted in the household setting by trained interviewers (dietitians) using a multi-component questionnaire. The questionnaire covered information on socio-demographic characteristics, household food security status, anthropometric measurements and dietary intake. Interviews lasted on average 45 min per household. Six interviewers were involved in data collection. Prior to the start of the study, all interviewers underwent a 5 day training workshop to ensure the standardization of data collection protocol and the validity and reliability of data collected. The first 4 days of this workshop offered a brief overview of the theoretical framework for the project as well as a series of extensive hands on activities addressing the various types of data collection (assessment of sociodemographic and household food security status, and collecting dietary intake and anthropometric measurements). On the fifth day, each interviewer independently collected data on a convenient sample of 5 households in the Greater Beirut area to pilot test the data collection protocol and tool. A post-field work meeting was held in the presence of the investigators. Based on the discussions during this meeting, the following modifications were introduced: 1) the order of questions was changed whereby first dietary intake was collected followed by the anthropometric measurements and finally the household food insecurity and sociodemographic sections were completed, 2) overall format of the questionnaire was revised to ensure clarity and compactness. In addition, throughout the data collection phase, weekly meetings were conducted to ensure the standardization of techniques and methods used. Results from the pilot testing were not included in the final analysis of the study.

The socio-demographic questions included mother’s age, number of children, income, crowding index, educational level of mother and spouse and their employment status (unemployed/employed). Crowding index is a commonly used measurement of household socio-economic status [28–30] that has been previously validated and used in Lebanon [15, 31, 32]. This index was calculated as the total number of co-residents per household divided by the total number of rooms, excluding the kitchen and bathrooms. Households with less than 2 persons per room were considered of lower crowding index compared to those with 2 or more persons per room.

HFIS in the present study was assessed using an Arabic translated and modified version of the Household Food Insecurity Access Scale (HFIAS) originally developed by the US Agency for International Development-funded Food and Nutrition Technical Assistance Project. The original HFIAS was previously validated in several contexts including the United States [33] and other LMICs such as Iran [20] and Tanzania [34]. In Lebanon, the adapted Arabic version of the HFIAS was also found to be a valid and reliable tool to assess HFIS showing high internal consistency (Cronbach’s alpha of 0.91) and reliability after being administered twice with an intra-class correlation coefficient of 0.58, *p* < 0.05 [11]. The Arabic version of the HFIAS was also tested for criterion-related validity and showed strong and consistent associations with various household characteristics, including total number of children in the household, family monthly income, crowding index, educational levels of mother and father, and number of cars. In addition, the same tool was found to be significantly associated with anthropometric measures [11]. This 9-question scale produces a total score between 0 and 27 with higher scores indicating greater food insecurity [33]. Households were categorised into four levels of food insecurity (food secure, mildly, moderately or severely food insecure) depending on the number of positive responses to questions related to severe conditions [33]. HFIS was later recoded into two variables (food secure vs food insecure) based on the HFIAS to explore its correlates and explore the association between HFIS and indicators of obesity.

Anthropometric assessment for study participants was conducted by trained dietitians using standard techniques and equipment. Participants were weighed on a digital scale to the nearest 0.1 kg wearing light clothing. Height was measured to the nearest 0.1 cm, without shoes. Waist circumference (WC) was measured to the nearest 0.1 cm using a plastic measuring tape at the midpoint between the bottom of the rib cage and the top of the iliac crest. Body mass index (BMI) (kg/m^2^) was calculated by dividing the weight (kg) over the height squared (m^2^). Obesity among mothers was measured in this study using two indicators, the BMI and at-risk WC. Mothers with a BMI ≥30 kg/m^2^ were classified as obese and those with a BMI less than 30 kg/m^2^ were considered to be non-obese, according to the Center for Disease Control and Prevention BMI classifications [35]. In addition, WC of women greater than or equal to 80 cm was classified as ‘at-risk WC’, and was used as a proxy measure for abdominal obesity, based on the International Diabetes Federation’s cut-points for Eastern Mediterranean and Middle East populations [36]. Elevated WC have been found to be associated with visceral adipose tissue [37], and it was used in the present study given the strong evidence that correlates abdominal fat with a higher risk of metabolic diseases, including diabetes, metabolic syndrome, and cardiovascular diseases [38, 39].

In the interview, dietary intake of mothers was assessed using the United States Department of Agriculture (USDA) multiple pass 24-h recall method [40]. Mothers were asked to recall foods and beverages that were consumed the day previous to the interview, or any other typical day. Two-D portion size posters and pictures were used to aid participants when estimating portion sizes of the reported foods and beverages (Millen and Morgan, Nutrition Consulting Enterprises, Framingham, Massachusetts, United States). These previously validated visuals have been well-accepted and commonly used in previous national studies conducted in Lebanon [41–43]. Daily energy, macronutrient and micronutrient intake of specific food groups were computed from the 24-h recalls using food composition database of the Nutritionist Pro software (version 5.1.0, 2014, SR 24, First Data Bank, Nutritionist Pro, Axxya Systems, San Bruno, CA). The food composition database was based on references from the USDA nutrient database and Lebanese specific foods and beverages were compared to culturally-specific food composition Tables [44]. Mean intake of 12 different food groups^1^ was calculated relative to total daily energy intake among the study sample to assess dietary quality. In addition, average nutrient intakes per day for food secure and food insecure mothers were calculated and percent of mothers consuming < 2/3rd the Dietary Reference Intakes (DRIs), recommended dietary allowance or adequate intake levels, for several nutrients were reported for both groups in the study [45].

### Statistical analysis

Descriptive statistics were reported for socioeconomic variables using means ± standard deviations and proportions with percentages for continuous and categorical variables, respectively. Crude and adjusted odds ratios were reported for associations between HFIS (food vs food insecure) and socio-demographic characteristics. The difference in mean intake of food groups and nutrients by HFIS was analysed using independent t-tests and reported using means and standard errors. In addition, the odds of consuming <2/3rd DRIs were explored among mothers in food insecure compared to food secure households, adjusting for energy. Multiple logistic regression models were conducted to explore the association of HFIS with obesity status (BMI ≥30 kg/m^2^) and at-risk WC (≥80 cm). These models were adjusted for variables found to be significant in the bivariate analyses. In case two variables were highly correlated (*P*-value <0.001), then one of the variables was excluded from the model to avoid multicollinearity. Results from logistic regressions were expressed as adjusted odds ratio (OR) with 95% confidence interval (CI). All data analyses were conducted using the Statistical Package for the Social and Sciences statistical software package (SPSS) version 22 with *p*-values of <0.05 considered statistically significant.

## Results

**Fig. 1:**
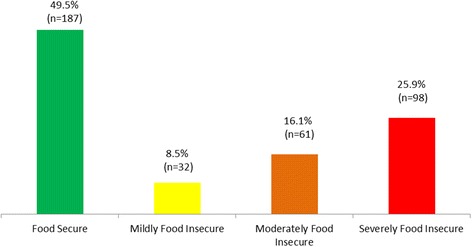
Prevalence of Household Food Insecurity in Beirut, Lebanon 2015 (*n* = 378)

Of the 388 households that completed the interviews, data from 378 households were included in the analysis after removing questionnaires with incomplete responses (response rate = 97%). Results presented in Fig. 1 showed that approximately 50% (95% CI: 44.5–54.5) of interviewed Lebanese households were food secure while 8% (95% CI: 5.7–11.3) were mildly food insecure, 16% (95% CI: 12.39–19.81) were moderately food insecure, and 26% (95% CI: 21.5–30.3) were severely food insecure.

Descriptive characteristics of mothers in the study sample by household food insecurity status and the associations between HFIS (food vs food insecure) and socio-demographic characteristics of households were presented in Table 1. Using bivariate analyses, HFIS was significantly associated with household income, crowding index, mother and spouse’s educational level and employment status (*P* < 0.01). After adjusting for other socio-demographic variables, household’s monthly income and mother’s education level remained significantly associated with HFIS. Food insecure households were significantly less likely to have a higher monthly income (*p* for trend <0.001) and to have a mother with higher levels of education (*p* for trend <0.01).

**Table 1:** Descriptive Characteristics of Mothers by Household Food Insecurity Status in Beirut, Lebanon, 2015 (*n* = 378)

	Total Sample (*n* = 378)	Food Secure (*n* = 187)	Food Insecure (*n* = 191)	Crude OR^a^ (95% CI)	Adjusted OR^b^ (95% CI)
Socio-demographic Characteristics					
	Mean ± SD			*OR (95% CI)*	
Number of Children	2.7 ± 1.1	2.7 ± 1.0	2.8 ± 1.2	1.09 (0.92, 1.31)	0.90 (0.70, 1.16)
Mother’s age (years)	39.7 ± 7.7	39.2 ± 7.5	40.3 ± 7.9	1.02 (0.99, 1.05)	1.03 (0.99,1.06)
	N (%)				
Household Monthly Income (L.L)^c^					
< 1,000,000 (reference)	119 (31.5)	21 (11.5)	98 (52.4)	1.0	1.0
1,000,000–2000,000	146 (38.6)	73 (39.9)	73 (39.0)	**0.21 (0.12, 0.38)**	**0.27 (0.15, 0.51)**
> 2000–0000	105 (28.4)	89 (48.6)	16 (8.6)	**0.04 (0.02, 0.08)**	**0.07 (0.03, 0.15)**
*p-for-trend*				**<0.001**	**<0.001**
Crowding Index					
< 2 person per room (reference)	330 (87.3)	175 (93.6)	155 (81.2)	1.0	1.0
> = 2 person per room	48 (12.7)	12 (6.4)	36 (18.8)	**3.39 (1.70, 6.74)**	2.18 (0.89, 5.32)
*p-for-trend*				**<0.001**	
Spouse’s Employment Status					
Unemployed (reference)	14 (3.8)	2 (1.1)	12 (6.6)	1.0	1.0
Employee/Self-employed	350 (96.2)	181 (98.9)	169 (93.4)	**0.16 (0.03, 0.71)**	0.33 (0.06, 1.67)
*p-for-trend*				**<0.01**	
Mother’s Employment Status					
Unemployed/ Homemaker (reference)	267 (70.8)	113 (60.8)	154 (80.6)	1.0	1.0
Employee/ Self-employed	110 (29.2)	73 (39.2)	37 (19.4)	**0.37 (0.23, 0.59)**	0.80 (0.43, 1.49)
*p-for-trend*				**<0.001**	
Spouse’s Education Level					
Intermediate school or less (reference)	188 (49.7)	66 (35.9)	122 (66.7)	1.0	1.0
High school or technical diploma	105 (27.8)	64 (34.8)	41 (22.4)	**0.35 (0.22, 0.57)**	0.60 (0.32, 1.11)
University degree	74 (19.6)	54 (29.3)	20 (10.9)	**0.20 (0.11, 0.36)**	0.92 (0.42, 2.04)
*p-for-trend*				**<0.001**	
Mother’s Education Level					
Intermediate school or less (reference)	161 (42.6)	50 (26.7)	111 (58.1)	1.0	1.0
High school or technical diploma	128 (33.9)	68 (36.4)	60 (31.4)	**0.40 (0.25, 0.64)**	**0.76 (0.42, 1.40)**
University degree	89 (23.5)	69 (36.9)	21 (10.5)	**0.13 (0.07, 0.24)**	**0.35 (0.16, 0.75)**
*p-for-trend*				**<0.001**	**<0.01**

*P*-values for entries that were in boldface and significant were added for binary variables including crowding index, spouse's employment status, and mother's employment status

*-Odds Ratio is significant at a p-value < 0.05.*

^a^ *Crude OR refers to unadjusted odds ratio of food insecurity among study sample*

^b^ *Adjusted OR refers to odds ratio of food insecurity after adjusting for all other socio-demographic variables*

^c^ *Currency rate: 1$ = 1500 L,L*

As presented in Table 2, 31% of mothers were obese (BMI ≥ 30 kg/m^2^) and 71% had an at-risk WC, reflecting central obesity. Significant differences were observed between food secure and food insecure households with respect to obesity and at-risk WC. Food insecure households had almost double the proportion of obese mothers compared to food secure ones (39.8% versus 21.4%) (*p* < 0.001). Moreover, a significantly higher proportion of mothers from food insecure households had at-risk WC compared to mothers in food secure ones (76.4% versus 64.7%, *p* = 0.012).

**Table 2:** Anthropometric measurements of Mothers by Household Food Insecurity Status in Beirut, Lebanon, 2015 (*n* = 378)

	Total Sample (*n* = 378)	Food Secure (*n* = 187)	Food Insecure (*n* = 191)	
	Mean ± SD			*P*-value^a^
Weight (kg)	70.9 ± 15.9	68.1 ± 13.5	73.7 ± 17.6	**0.001**
Height (cm)	158.6 ± 7.1	159.7 ± 6.1	157.6 ± 7.9	**0.004**
Body Mass Index (BMI), n(%)				
Normal Weight	134(35.4)	84(44.9)	50(26.2)	**0.000**
Overweight	128(33.9)	63(33.7)	65(34.0)	
Obese	116(30.7)	40(21.4)	76(39.8)	
Waist Circumference (WC), n(%)				
Normal WC <80 cm)	111(29.4)	66(35.3)	45(23.6)	**0.012**
At-Risk WC (≥80 cm)	267(70.6)	121(64.7)	146(76.4)	

^a^Significantly different at *p*-value <0.05; *p*-value was derived using t-test for continuous variables and chi-square analysis for categorical variables

Differences in food consumption of mothers in food secure compared to food insecure households were also observed. Table 3 displays the percent of daily energy intake from 12 different food groups by food insecurity status. It was noticed that mothers from food insecure households had a significantly higher percent daily energy intake from bread & cereals, eggs, and sweets & sugar-sweetened beverages compared to mothers from food secure ones (*p* < 0.038). However, the percent daily energy intake from nuts, dairy products, fruits, and alcoholic beverages was significantly higher among mothers from food secure compared to insecure households (*p* < 0.034).

**Table 3:** Mothers’ percent daily energy intake from various food groups by household food security status (*n* = 378)

	Food Secure (*n* = 187)	Food Insecure (*n* = 191)	
Food Groups	Mean % of Daily Energy Intake ± SE		*P*-value*
Bread & Cereals	31.2 ± 1.2	35.0 ± 1.4	**0.038**
Starchy Vegetables & Legumes	3.4 ± 0.5	3.5 ± 0.6	0.922
Nuts	2.92 ± 0.4	0.93 ± 0.3	**0.009**
Seeds	0.89 ± 0.3	1.46 ± 0.4	0.258
Dairy Products	11.29 ± 0.9	8.05 ± 0.8	**0.006**
Eggs	0.35 ± 0.1	1.11 ± 0.3	**0.026**
Meat, Poultry, Fish	11.4 ± 1.0	9.7 ± 0.9	0.207
Fruits	7.81 ± 0.7	5.80 ± 0.6	**0.030**
Vegetables	3.70 ± 0.3	3.41 ± 0.4	0.548
Added Oils	14.49 ± 0.9	14.65 ± 0.9	0.904
Sweets & Sugar Sweetened Beverages	9.9 ± 0.7	12.9 ± 1.1	**0.026**
Alcoholic Beverages	0.99 ± 0.4	0.18 ± 0.1	**0.034**

*Significantly different at *p-*value <0.05

Average daily energy, macro- and micronutrient intakes and the odds of consuming less than 2/3rd the DRIs for individual nutrients were compared between mothers from food secure and food insecure households, after adjusting for energy. Significant differences were observed among mothers from both groups with respect to their intake of key micro-nutrients, as displayed in Table 4. Compared to mothers in food secure households, mothers in food insecure households had significantly lower mean daily intakes of key micronutrients, including calcium, potassium, and vitamin C. Additionally, a significantly higher percentage of mothers in food insecure households than food secure ones was consuming less than 2/3^rds^ the DRIs for potassium, folate, vitamin C, and vitamin B6 (*p* < 0.05), after adjusting for energy. With respect to total daily energy intake, no significant difference was observed between mothers from food insecure and secure households (1246 ± 55 kcal vs 1286 ± 46 kcal per day, respectively; data not shown). As for macronutrient intake, it was found that mean daily intake of protein was significantly lower among mothers in food insecure than food secure households (41 ± 2 g vs 47 ± 2 g per day, respectively). Daily total fat intake was also on average lower among mothers from food insecure households compared to food secure ones (53 ± 3 g vs 56 ± 2 g respectively), whereas mean intake of carbohydrates was higher among mothers from food insecure households versus food secure ones (156 ± 8 g per day vs 149 ± 6 g per day). Differences in total fat and carbohydrate intakes among mothers from food secure and insecure households were not found to be statistically significant (data not shown). Although a higher proportion of obese mothers in this study (using BMI and at-risk waist circumference) had inadequate micronutrient intakes compared to non-obese mothers, differences between both groups did not reach statistical significance (data not shown).

**Table 4:** Nutrient Intakes among Mothers by Household Food Insecurity Status in Beirut, Lebanon, 2015 (*n* = 378)

	*DRIs*	*Intake/day* *Mean* ± SE	*% Consuming* *(<2/3 DRIs)* ^c^	*OR* ^d^ *(95% CI)*
Calcium (mg)	1000–1300 mg			
Food Secure		**532.6 ± 28.9** ^a^	72.7	1.00
Food Insecure		**416.5 ± 22.7** ^b^	83.8	1.82 (0.82–4.06)
Sodium (mg)	1200–1500 mg			
Food Secure		1536.4 ± 71.5	35.8	1.00
Food Insecure		1513.1 ± 78.0	38.2	0.96 (0.58–1.57)
Potassium (mg)	4700 mg			
Food Secure		**1777.0 ± 72.1** ^a^	90.4	1.00
Food Insecure		**1503.6 ± 79.2** ^b^	93.2	**2.01 (1.23–3.29)**
				**0.005**
Iron (mg)	8–18			
Food Secure		8.8 ± 0.4	77.5	1.00
Food Insecure		8.6 ± 0.5	77.5	0.70 (0.34–1.45)
Zinc (mg)	*9–8 mg*			
Food Secure		5.5 ± 0.3	57.2	1.00
Food Insecure		5.5 ± 0.3	61.8	0.89 (0.49–1.61)
Folate (μg)	*400* μg			
Food Secure		231.2 ± 15.1	71.1	1.00
Food Insecure		192.0 ± 13.3	78.5	**2.12 (1.04–4.33)**
				**0.038**
Phosphorous (mg)	700–1250 mg			
Food Secure		647.0 ± 25.0	33.7	1.00
Food Insecure		585.6 ± 31.2	47.1	1.60 (0.93–2.76)
Magnesium (mg)	310–360 mg			
Food Secure		187.8 ± 7.7	66.3	1.00
Food Insecure		169.5 ± 9.6	78.5	1.32 (0.61–2.84)
Selenium (μg)	55 μg			
Food Secure		55.1 ± 3.0	35.3	1.00
Food Insecure		50.2 ± 2.5	40.8	1.00 (0.61–1.61)
Vitamin C (mg)	65-75 mg			
Food Secure		**66.9 ± 4.8** ^a^	52.9	1.00
Food Insecure		**53.6 ± 4.7** ^b^	67.0	**1.59 (1.00–2.53)**
				**0.049**
VitaminA (μg)	700 μg			
Food Secure		571.8 ± 42.4	55.1	1.00
Food Insecure		482.8 ± 40.7	63.9	1.24 (0.77–1.99)
Vitamin D(μg)	15–20 μg			
Food Secure		0.8 ± 0.1	99.5	---
Food Insecure		0.8 ± 0.2	98.4	---
Vitamin B1 mg	1.0–1.1 mg			
Food Secure		0.8 ± 0.0	49.2	1.00
Food Insecure		0.8 ± 0.0	55.8	1.58 (0.86–2.93)
VitaminB2 (mg)	1.0–1.1 mg			
Food Secure		0.895 ± 0.04	43.8	1.00
Food Insecure		0.829 ± 0.04	52.6	1.28 (0.75–2.20)
Vitamin B3 (mg)	14 mg			
Food Secure		12.2 ± 0.7	47.3	1.00
Food Insecure		10.7 ± 0.6	53.4	1.41 (0.84–2.39)
VitaminB6 (mg)	1.2–1.5			
Food Secure		1.0 ± 0.0	50.8	1.00
Food Insecure		0.9 ± 0.1	64.4	**2.05 (1.15–3.65)**
				**0.015**
Vitamin B12 (μg)	2.4μg			
Food Secure		6.0 ± 2.6	66.8	1.00
Food Insecure		4.6 ± 1.5	68.4	0.90 (0.55–1.48)

^*a,b*^ *Values with different superscripts are significantly different p < 0.05 using T Test for comparison of mean intake per day and percentage of subjects consuming less than two thirds of the recommendations*

^c^ *Dietary Reference Intake (DRI) refers to the Recommended Daily Intake (RDA) and Adequate Intake (AI) for nutrients as recommended by the Institute of Medicine* [45]

^d^ *Odds ratio was adjusted for total daily energy intake*

Logistic regression analyses showed that mothers in food insecure households were significantly more likely to be obese compared to mothers in food secure households (unadjusted OR = 2.43; 95% CI: 1.54–3.82). This association remained significant (adjusted OR = 1.73; 95% CI: 1.02–2.92), even after adjusting for potential confounding variables, including number of children in the household, mother’s age, crowding index, educational level and employment status of mothers and their spouses. Household income was excluded from the model to avoid multicollinearity, as it was found to be significantly associated with crowding index (chi-square = 22.9, *P*-value <0.001). In addition, data on the crowding index of households was found to be complete in this study and may have been subject to less risk of reporting bias compared to income, as reported in other studies [46–48]. Furthermore, mothers from food insecure households were significantly more likely to have an at-risk WC (unadjusted OR = 1.77; 95% CI: 1.13–2.77). However, this association was no longer statistically significant after adjusting for socio-demographic variables (see Table 5).

**Table 5:** Association of Obesity with Household Food Insecurity Status Among Mothers in Beirut, Lebanon 2015 (*n* = 378)

	Obesity (BMI ≥30 kg/m^2^)	At-Risk WC (≥80 cm)
	OR (95% CI)	OR (95% CI)
Model 1 (Unadjusted)^a^		
Household food insecurity		
Food Secure (*Reference)*	1.0	1.0
Food Insecure	**2.43 (1.54–3.82)**	**1.77 (1.13–2.77)**
Model 2 (Adjusted)^b^		
Household food insecurity		
Food Secure (*Reference)*	1.0	1.0
Food Insecure	**1.73 (1.02–2.92)**	1.48 (0.87–2.50)

^a^ *Model 1 is unadjusted. Crude odds ratios are reported for obesity status (BMI ≥ 30 kg/m2) and at-risk WC (≥80 cm*

^b^ *Model 2 is adjusted for the following variables (number of children in household, mother’s age, crowding index, spouse’s employment status, mother’s employment status, spouse’s educational level, and mother’s educational level). Adjusted odds ratios are reported for obesity and at-risk* WC

## Discussion

This is the first study in Lebanon and the MENA region to explore the prevalence and correlates of experience-based HFIS among a representative sample of urban Lebanese households and to examine whether HFIS is associated with the increased risk of obesity and dietary inadequacies among mothers from the study sample. Findings from the present study show that almost 50% of interviewed households reported food insecurity with 42% suffering from moderate to severe food insecurity and 8% living with mild food insecurity. The prevalence of moderate to severe food insecurity among Beirut residents was found to be higher than that reported earlier by rural households in the Bekaa region and another semi-urban Southern Lebanese population (34% and 42%, respectively) [11, 12]. However, comparable food insecurity levels to those reported in Greater Beirut area were observed among urban communities within LMICs with rates ranging between 34.4% in Ho Chi Min City, Vietnam [49] to 74.6% in an urban area in Tamil Nadu, India [50] and 81% among urban households in Quito, the 2nd largest city in Ecuador [51].

Food insecurity was historically considered a rural phenomenon, particularly in LMICs [52]. However, recent studies report that urbanisation may contribute to food and nutrition insecurity, particularly in cash-based economies where urban and suburban households are primarily dependent on a stable access to income to ensure their health and well-being [53]. In Lebanon, the urban expansion has been witnessed in all of its major cities including Beirut, the capital of the country, with 98% of its total area reported to be urbanised [54]. This urban sprawl has lead over the years to overpopulation and transformed open spaces within certain suburbs of the city into large slums that worsened the living conditions and increased poverty rates among many of its residents [55]. Concomitantly, urbanisation has been linked to major shifts in the food landscape and to changes in food consumption, physical activity and other lifestyle behaviors of adults and children. These changes, also known as the nutrition transition, are associated with increasing rates of overweight and obesity and to heightened risk of developing chronic diseases [56, 57].

In parallel to the urbanisation phenomena, Lebanon, as with other LMICs in the MENA region, has been undergoing dramatic economic and social challenges due to the ongoing political and military conflicts post the *Arab Spring* leading more than a million families to seek refuge within the borders of the country. The recent refugee estimates, according to the United Nations High Commissioner for Refugees (UNHCR), exclude Palestinian refugees who have been in Lebanon for more than five decades and refugees who have fled Iraq since the 2003 invasion and the Persian-Gulf war in the early 1990’s. Given the high numbers of refugees and the heightened challenges accompanying their displacement to Lebanon, several studies have been published exploring HFIS among refugee groups, however this study is the first to explore correlates of HFIS among a representative sample of urban Lebanese households. Rates of moderate to severe HFIS reported in the Greater Beirut area (42%) were found to be surprisingly higher than those reported in Beirut among Syrian refugees in 2015 and 2016 (19% and 22%, respectively), yet lower than those reported in previous studies among Palestinian and Iraqi refugees [58, 59]. The high prevalence of moderate and severe HFIS among Lebanese households and refugees who have been in Lebanon for extended periods of time can be attributed in part to the insufficient welfare programs that address the increasing needs of these communities, particularly those residing in impoverished suburbs of the city, in comparison to the large-scale international aid and humanitarian assistance provided to the recently displaced Syrian refugees. The difference in severity of experience-based food insecurity can be also explained in part by discrepancies in perceptions due to increased social tension and competition between refugee groups and the host community on limited livelihood opportunities, such as food, jobs, decent accommodation, public health, and educational services [60, 61]. All of these factors may have contributed to the high risk of food insecurity reported among Lebanese host communities in Beirut [61].

Socioeconomic correlates of HFIS reported in this study were found to be similar to those reported in the scientific literature [62–64]. Indicators of low socioeconomic status, such as low income and high crowding index, as well as low educational levels of mothers and their spouses were found to be significantly associated with HFIS and some of these variables remained significant event after adjusting for other socioeconomic correlates.

With respect to dietary intake, results from the present study showed different food consumption patterns and nutrient intakes among mothers from food secure versus food insecure households. Food insecure mothers had on average a significantly higher percent daily energy intake from breads & cereals, eggs, and sweets & sugar-sweetened beverages as well as a significantly lower intake of nuts, dairy products, and fruits compared to food secure mothers. However, no significant differences were observed in terms of daily energy intake among mothers from both food secure and food insecure households. The lack of significance could be attributed to the overall low energy intake reported among mothers from both groups. This low energy intake may be explained by a number of factors, including underreporting that is commonly identified among women [65] particularly those that are overweight or obese [66, 67]. In addition, the use of only one 24-h recall method to assess dietary intake of mothers may have contributed to the low energy intake assessed in this study among both groups [68]. Thus, these factors may have masked potential differences in total energy intake between mothers from food secure versus insecure households. Nevertheless, macronutrient intake of mothers was shown to differ based on HFIS. It was noted that mothers from food insecure households had significantly lower intake of proteins compared to mothers from food secure households. In addition, carbohydrate intake was higher among food insecure mothers, even though, this difference did not reach statistical significance. These findings are in line with previous studies conducted in LMICs whereby women from food insecure households were at increased risk of low dietary diversity characterised by lower intake of animal-based foods that are rich in proteins, including dairy products, meats, and fish, compared to women from food secure households [69, 70]. In addition, studies conducted in Brazil and Mexico reported negative associations between food insecurity with intakes of fruits, vegetables, and protein foods (meat/fish/dairy) amongst not only mothers [71], but also children [72]. In fact, similar differences in dietary intakes have been reported among different refugee groups in Lebanon whereby food insecure households relied more on cereals, fats, and sweets within their regular diet compared to more expensive foods such as meats, dairy products, and fruits [59, 73]. Thus, HFIS may increase the consumption of low-cost energy-dense diets, which in turn can increase the risk of weight gain and obesity [13, 24, 74] and other associated co-morbidities, including type 2 diabetes and hypertension among adult women [13].

Reported sacrifices in dietary diversity can have serious implications not only on the risk of obesity, but also on the micronutrient intake of women, as seen in the present study. Adjusting for energy, food insecure mothers were found to be significantly more likely than their food secure counterparts to consume less than 2/3rd the DRIs for key nutrients, including potassium, folate, vitamin C, and vitamin B6. This level of intake is generally accepted as minimally adequate for individuals according to previous studies conducted in Lebanon [75, 76]. The consumption of low nutrient, energy-dense foods such as breads and cereals, sugars & sugar-sweetened beverages among mothers from food insecure households may have contributed to their increased risk of nutrient deficiencies. Similar higher risks of nutrient inadequacies, including protein, folate, vitamin C, and vitamins B6 were reported among women of reproductive age from food insecure households in Canada and the United States [77–79]. Limited studies have been published to date reporting nutrient inadequacies among food insecure women of reproductive age [25] and female caregivers and their children within urban settings in LMICs [26]. Differences in dietary intakes according to food insecurity status may be explained by the different coping mechanisms adopted by mothers to protect their children from hunger at the expense of their own dietary intake in terms of quantity and adequacy [2–4]. Indeed, studies have shown that food insecurity adversely affects the quality and variety of diets consumed by women [63, 80]. Mothers in food insecure households may limit their food intake, skip meals, or consume affordable yet low nutrient-dense diets to limit their feelings of hunger while ensuring that their children are being well-fed [5]. Other reasons that could explain the dietary differences between mothers in food secure and insecure households could be attributed to the high consumption of breads and cereals among lower income households. Although bread is rich in carbohydrates, it is deficient in numerous micronutrients, including folate, potassium, zinc, vitamins A, C, D, and E [44, 81].

Findings from the present study demonstrate a significant association between HFIS and obesity (BMI ≥ 30 kg/m^2^) among mothers. The positive association between food insecurity and obesity markers has been well-established among women in high income countries [5, 19, 62, 80], however, the evidence is less conclusive in LMICs and those undergoing nutrition transitions [82, 83]. Differences in findings across studies could be explained by the stage of nutrition transition undergone in each of the contexts of these studies, which may in turn affect the food consumption and lifestyle behaviors of women. Another reason could be the different criteria used by researchers to measure abdominal obesity. For example, Mohammadi and colleagues [83] reported a 2.05 higher risk of overweight among moderately food insecure women and 2.8 times higher risk of abdominal obesity (using the North American cut-point of WC ≥88 cm) among severely food insecure households compared to their food secure counterparts in Tehran city (Iran). Another study conducted in Malaysia among 200 women from rural communities showed a similar significant association between food insecurity and at-risk WC (using the 88 cm cut-off), but not overweight status (BMI ≥ 25 kg/m^2^), after adjusting for a number of covariates [21]. Whereas in the present study, the cut point of ≥80 cm was used for Lebanese women, as recommended by the IDF criteria for Eastern Mediterranean and Middle Eastern populations [36]. In fact, the same cutoff-points that are based on European data have been recommended for Sub-Saharan African and South Asian populations (including Malaysian and Chinese) in the absence of specific data for these ethnic groups.

It is worth noting that a significant association was also observed between HFIS and at-risk WC in the present study, however this association was no longer significant after adjusting for socioeconomic correlates. Maternal education, one of the socioeconomic factors adjusted for in this study, has been associated with healthier eating and lifestyle patterns that may have been protective against obesity [21, 84, 85]. This may explain why women from food insecure households compared to their food secure counterparts did not exhibit an elevated WC, which is a proxy measure, for visceral fat. In fact, a previous study conducted in Lebanon have shown that female gender and having a higher education were positively associated with odds of metabolically healthy overweight and obesity, a phenotype that is characterized with a favorable metabolic profile such as insulin sensitivity, normal blood pressure and lipid profile [86]. In addition, a negative association was observed between being metabolically healthy overweight/obese and waist circumference in that study, which may be another protective factor from visceral fat accumulation among women in the present study.

Although studies report that food insecurity and obesity may coexist among women within disadvantaged households from high and LMICs [20, 24, 49, 62]; the mechanisms for this paradoxical association are still not well-elucidated. Research proposed a number of hypotheses and mechanisms to explain this association. One proposed explanation refers to evidence showing that adults living in poverty can have adequate caloric intake or even levels that exceed their energy requirements; however the quality of their diets may be compromised due to the absence of diversity and nutrient adequacy [7]. The regular consumption of such diets can lead to the double burden of malnutrition, whereby women, may suffer from obesity coupled with micronutrient deficiencies. The ‘sacrifice theory’ is another proposed mechanism suggesting that mothers tend to adopt unfavorable coping strategies, including sacrificing their own dietary intake and nutritional health, for the sake of their children [19, 20, 87]. Moreover, to manage feeding their families on limited budgets, mothers may skip meals and consume diets of low diversity that are calorically-dense yet of low nutritional value, which may in turn increase their risk of weight gain [80]. Other researchers argue that food insecure women may undergo cyclical eating patterns whereby they can go for extended periods of time with severe unintentional food restrictions followed by overeating episodes to compensate when food is available in abundance [19, 83]. This continuous ‘feast or famine’ situation during periods of food shortage and abundance may lead to metabolic changes and contribute to gradual weight gain over time [83]. Maternal stunting during childhood has also been proposed as an underlying mechanism for the association between HFIS and obesity due to changes in the energy metabolism among mothers who experience stunting during early stages of life [82]. Other factors that can explain this paradoxical relationship are related to increased exposure to mental, physical, and psychological stressors among poor and food insecure mothers, which may contribute to a higher risk of depression, increased sedentary behaviors, and other potential adverse health behaviors that are related to weight gain [19, 20, 88].

Findings from this study need to be interpreted in light of a number of limitations. First, the dietary intake of mothers was explored using only one 24-h recall per participant, which could have contributed to the overall low energy intake reported among women in this study. Another reason could be social desirability bias, whereby survey participants could have responded in a manner that they perceived as acceptable or favorable to the interviewer [89]. However, every attempt was exerted to minimize any social desirability bias, whereby interviewers who conducted data collection received extensive training to reduce judgmental verbal and non-verbal communication. In addition interviewers were trained dietitians who underwent a 5-day training workshop to ensure the standardization of data collection protocol and the validity and reliability of collected data and meetings were scheduled throughout the data collection phase of the study to ensure the standardization of techniques and methods. Furthermore, interviewers followed the 5 steps of the USDA multiple pass 24-h recall method to increase the accuracy of dietary assessment which include (1) the quick list; (2) the forgotten foods list; (3) time and occasion at which foods were consumed; (4) the detail cycle; and (5) the final probe review. Data was also collected on weekdays and weekends to ensure that dietary data represents usual intake among mothers. Another limitation worth mentioning is the design of the study, which is cross-sectional in nature, limiting our ability to explore the causal pathways between HFIS, risk of dietary inadequacies, and obesity among mothers. Nevertheless, results from the present study can provide baseline data for future trend analyses and comparisons regarding the prevalence and covariates of food security status and its implications on health and nutritional outcomes of women.

## Conclusions

In conclusion, this study was the first to explore the associations between experience-based household food insecurity and risk of dietary inadequacies and obesity among mothers from a representative sample of urban households in a LMIC within the MENA region. High HFIS was reported among Lebanese households. In addition, mothers from food insecure households were at an elevated risk of suffering from dietary inadequacies and obesity, which in turn can increase the risk of diet-related diseases and may lead to undesirable nutritional and health outcomes among mothers and their offspring. Thus, adequate intervention programmes need to be developed and implemented to support sustainable livelihood opportunities for at-risk population groups, including women, within urban settings that are also enduring the undesirable repercussions of the high refugee influx into their communities. In addition, findings from this study can provide policy makers and public health professionals with evidence to devise and implement culture and context-specific interventions that can help improve the nutrition and health of mothers. More specifically, programs can be developed at the local and national level to increase the dietary quality and diversity of foods consumed by women and mothers through promoting the consumption of affordable nutrient-dense foods including fruits, vegetables, and protein-rich foods, and lowering the intake of added sugars and refined grains. However, efforts need to be exerted to test the effectiveness of these strategies and other potential approaches in reducing the vulnerability of mothers to the adverse consequences of food insecurity including the burdens of nutrient insecurity and obesity, particularly in LMICs undergoing rapid nutrition transition.

## Abbreviations

AMDR: acceptable macronutrient distribution range
BMI: body mass index
CI: confidence interval
DRI: dietary reference intakes
FAO: Food and Agriculture Organization
HFIAS: Household Food Insecurity Access Scale
HFIS: household food insecurity
IDF: International Diabetes Federation
IOM: Institute of Medicine
LMIC: low and middle income countries
MENA: Middle East and North Africa
OR: odds ratio
USDA: United States Department of Agriculture
WC: waist circumference
WHO: World Health Organization

## Footnotes

1 The following food groups, as presented in Table 3, were analysed: Bread and cereals, starchy vegetables and legumes, nuts, seeds, dairy products (including milk, yoghurt, cheese, and labneh, strained yoghurt that is commonly consumed within this population), eggs, meat/poultry/fish, fruits, vegetables, added oils, sweets and sugar sweetened beverages (such as cookies, candy, chocolate, carbonated beverages, and local Arabic sweets) and alcoholic beverages.
